# Supplementary material for: The Small RNA Universe of Capitella teleta
Source: Front Mol Biosci. 2022 Feb 25;9:802814. doi: 10.3389/fmolb.2022.802814 (PMC8915122; doi:10.3389/fmolb.2022.802814)
Supplement: Supplementary file 1 [file DataSheet1.ZIP › Supplement/known/CAPTEscaffold_292_14539.pdf]

Provisional ID	:	CAPTEscaffold_292_14539
Score total	:	4.5
Score for star read(s)	:	-1.3
Score for read counts	:	0
Score for mfe	:	1.2
Score for randfold	:	1.6
Score for cons. seed	:	3
Total read count	:	265874
Mature read count	:	265856
Loop read count	:	0
Star read count	:	18

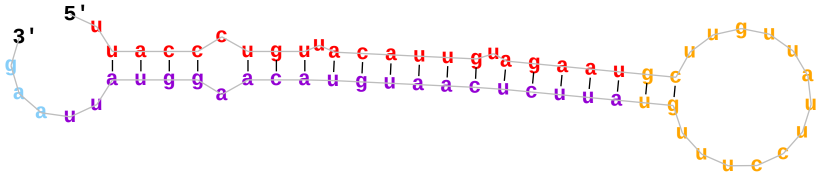

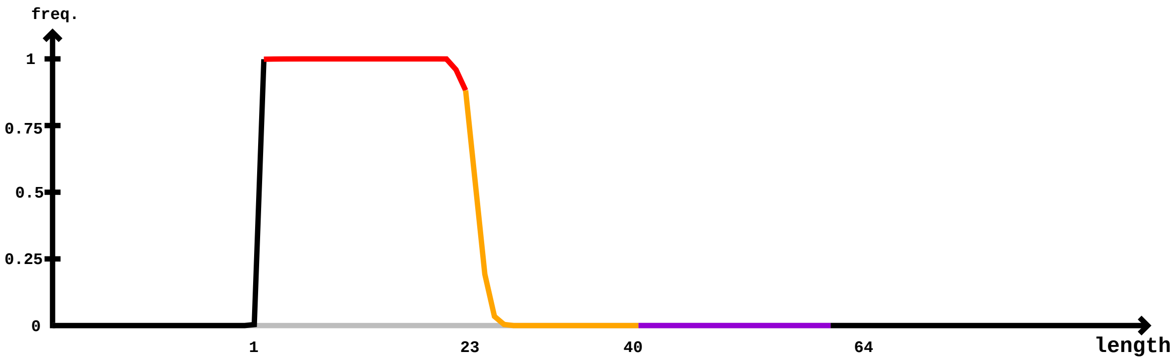


```
                  Mature                                 Star
5'- caauccucgccaucaauucuuuacccuguuacauuguagaaugcuuguuauuccuuuguauucucaauguacaagguauuaagaauuggagguugaauuacuuccggaaguc  -3'  obs
    caauccucgccaucaauucuuuacccuguuacauuguagaaugcuuguuauuccuuuguauucucaauguacaagguauuaagaauuggagguugaauuacuuccggaaguc       exp
    caauccucgccaucaauucuuuacccuguuacauuguagaaugcuuguuauuccuuuguauucucaauguacaagguauuaagaauuggagguugaauuacuuccggaaguc       known
    ...((((((((.(((((((((((((.(((.((((((.(((((((.............))))))))))))))))).)))).)))))))))).)).))).........))).... reads   mm   sample

    ...................uuuacccuguuacauuguaga........................................................................ 38      0    seq
    ...................uAuacccuguuacauuguagaa....................................................................... 1       1    seq
    ...................uuuacccuguuacauuguagaa....................................................................... 11      0    seq
    ...................Cuuacccuguuacauuguagaa....................................................................... 1       1    seq
    ...................uuuacccuguuUcauuguagaau...................................................................... 1       1    seq
    ...................uuuacccuguuacauuguagaau...................................................................... 499     0    seq
    ...................Cuuacccuguuacauuguagaau...................................................................... 6       1    seq
    ...................uAuacccuguuacauuguagaau...................................................................... 1       1    seq
    ...................uuuacAcuguuacauuguagaau...................................................................... 1       1    seq
    ...................Auuacccuguuacauuguagaau...................................................................... 1       1    seq
    ...................uuuacccuguuacauuguagaaA...................................................................... 17      1    seq
    ...................uuuacccuguuacauuguagaaug..................................................................... 198     0    seq
    ...................Cuuacccuguuacauuguagaaug..................................................................... 8       1    seq
    ...................Auuacccuguuacauuguagaaug..................................................................... 1       1    seq
    ...................uuuaccUuguuacauuguagaaug..................................................................... 1       1    seq
    ...................uuuacccuguuacauuguagaaugU.................................................................... 12      1    seq
    ...................Cuuacccuguuacauuguagaaugc.................................................................... 29      1    seq
    ...................uuuacccuguuacauAguagaaugc.................................................................... 2       1    seq
    ...................uuuacccuguuacauuguagaaugc.................................................................... 138     0    seq
    ...................Auuacccuguuacauuguagaaugc.................................................................... 1       1    seq
    ...................uuuacccuguuacauuguagaaugA.................................................................... 1       1    seq
    ...................Cuuacccuguuacauuguagaaugcu................................................................... 1       1    seq
    ...................uuuacccuguuacauuguagaaugcu................................................................... 2       0    seq
    ...................uuuacccuguuacauuguagaaugcA................................................................... 31      1    seq
    ....................uuacccuguuacauuguag......................................................................... 67      0    seq
    ....................uuacccuguuAauuguaga........................................................................ 2       1    seq
    ....................uuacccuguuacauuguagG........................................................................ 5       1    seq
    ....................Guacccuguuacauuguaga........................................................................ 2       1    seq
    ....................uuacccuguuacauuguaAa........................................................................ 3       1    seq
    ....................Auacccuguuacauuguaga........................................................................ 47      1    seq
    ....................uuacUcuguuacauuguaga........................................................................ 5       1    seq
```

```
                    Mature                                 Star
caauccucgccaucaauucuuuacccuguuacauuguagaaugcuuguuauuccuuuguauucucaauguacaagguauuaagaauuggagguugaauuacuuccggaaguc
....................uuacccuguuGcauuguaga........................................................................    4      1    seq
....................uuacccuguuacauugAaga........................................................................    4      1    seq
....................uuacccuguuacauuguUga........................................................................    3      1    seq
....................uuacccuguuacUuuguaga........................................................................    1      1    seq
....................uuacccuguuacaAuguaga........................................................................    1      1    seq
....................uuaUccuguuacauuguaga........................................................................    2      1    seq
....................uuacccuguuacauAguaga........................................................................    1      1    seq
....................uuacccuguuacauuguagU........................................................................    1      1    seq
....................uuacccugAuacauuguaga........................................................................    2      1    seq
....................uuacccuguuacauuAuaga........................................................................    3      1    seq
....................uuacccuguuacauuguGga........................................................................    4      1    seq
....................uuacccuguuacaCuguaga........................................................................    25     1    seq
....................uuacccuguuacauugCaga........................................................................    1      1    seq
....................uuacccuguuacauuguaUa........................................................................    2      1    seq
....................uuaccUuguuacauuguaga........................................................................    4      1    seq
....................uuacccuguuacaGuguaga........................................................................    1      1    seq
....................uuacccuguuacauuguaCa........................................................................    1      1    seq
....................uuaAccuguuacauuguaga........................................................................    4      1    seq
....................uuacccAguuacauuguaga........................................................................    6      1    seq
....................uuacccuguuacGuuguaga........................................................................    1      1    seq
....................uuacccuguuacauuUuaga........................................................................    1      1    seq
....................uuGcccuguuacauuguaga........................................................................    2      1    seq
....................uuacccuguuUcauuguaga........................................................................    1      1    seq
....................uuacccuguuaUauuguaga........................................................................    2      1    seq
....................uuacAcuguuacauuguaga........................................................................    3      1    seq
....................uuacccuguAacauuguaga........................................................................    5      1    seq
....................uAacccuguuacauuguaga........................................................................    9      1    seq
....................uuaGccuguuacauuguaga........................................................................    1      1    seq
....................uuacccuguuacauuguaga........................................................................    10425  0    seq
....................uuacccuguuacauGguaga........................................................................    1      1    seq
....................uuaccAuguuacauuguaga........................................................................    8      1    seq
....................uCacccuguuacauuguaga........................................................................    2      1    seq
....................uuacccuAuuacauuguaga........................................................................    3      1    seq
....................uuacccuguuacauAguagaa.......................................................................    1      1    seq
....................uAacccuguuacauuguagaa.......................................................................    16     1    seq
....................Auacccuguuacauuguagaa.......................................................................    87     1    seq
....................uuacccuguuacauuguagUa.......................................................................    4      1    seq
....................uuaUccuguuacauuguagaa.......................................................................    2      1    seq
....................uuacccuguuacauuguagGa.......................................................................    2      1    seq
....................uuGcccuguuacauuguagaa.......................................................................    3      1    seq
....................uuacccGguuacauuguagaa.......................................................................    2      1    seq
....................uuacccuguuacauuguagaa.......................................................................    20271  0    seq
....................uuacccugGuacauuguagaa.......................................................................    1      1    seq
....................uuacccuguuacaCuguagaa.......................................................................    2      1    seq
....................uuacccAguuacauuguagaa.......................................................................    14     1    seq
....................uuacccuguuaAauuguagaa.......................................................................    5      1    seq
....................uuacccugAuacauuguagaa.......................................................................    6      1    seq
....................uuacccuguCacauuguagaa.......................................................................    2      1    seq
....................uuacccuguuacauuguUgaa.......................................................................    2      1    seq
....................uuacccuAuuacauuguagaa.......................................................................    4      1    seq
....................uuacUcuguuacauuguagaa.......................................................................    7      1    seq
....................uuUcccuguuacauuguagaa.......................................................................    1      1    seq
....................uuacccuguuacauugAagaa.......................................................................    8      1    seq
....................uCacccuguuacauuguagaa.......................................................................    3      1    seq
....................uuacccuguuacauuguGgaa.......................................................................    3      1    seq
....................uGacccuguuacauuguagaa.......................................................................    1      1    seq
....................uuacccuguuacauuAuagaa.......................................................................    1      1    seq
....................uuacccuguuacauCguagaa.......................................................................    3      1    seq
....................Cuacccuguuacauuguagaa.......................................................................    1      1    seq
....................uuacccCguuacauuguagaa.......................................................................    1      1    seq
....................uuacccuguuacauuguagaU.......................................................................    4      1    seq
....................uuacccuguAacauuguagaa.......................................................................    7      1    seq
....................uuacccuguNacauuguagaa.......................................................................    1      1    seq
....................uuacccuguuGcauuguagaa.......................................................................    1      1    seq
....................uuacccuguuacGuuguagaa.......................................................................    8      1    seq
....................uuacccuguuacauuCuagaa.......................................................................    2      1    seq
....................uuacccuguuacUuuguagaa.......................................................................    5      1    seq
....................uuaccAuguuacauuguagaa.......................................................................    17     1    seq
....................uuacccuguuacCuuguagaa.......................................................................    1      1    seq
....................uuaccUuguuacauuguagaa.......................................................................    16     1    seq
```

```
                    Mature                                     Star
caauccucgccaucaauucuuuacccuguuacauuguagaaugcuuguuauuccuuuguauucucaauguacaagguauuaagaauuggagguugaauuacuuccggaaguc
....................uuacccuguuacauuguaAaa.......................................................................   7        1    seq
....................uuacccuguuCcauuguagaa.......................................................................   1        1    seq
....................uuacccuguuacaAuguagaa.......................................................................   5        1    seq
....................uuacccugCuacauuguagaa.......................................................................   5        1    seq
....................uuacccuguuUcauuguagaa.......................................................................   1        1    seq
....................uuacccuguuacaGuguagaa.......................................................................   1        1    seq
....................uuacccuguuacauuUuagaa.......................................................................   2        1    seq
....................Guacccuguuacauuguagaa.......................................................................   5        1    seq
....................uuacccuguuacauuguagaG.......................................................................   3        1    seq
....................uuacccuguuacauugCagaa.......................................................................   1        1    seq
....................uuacAcuguuacauuguagaa.......................................................................   26       1    seq
....................uNacccuguuacauuguagaa.......................................................................   1        1    seq
....................uuaAccuguuacauuguagaa.......................................................................   6        1    seq
....................uuacGcuguuacauuguagaa.......................................................................   2        1    seq
....................uuacccuguuacauuguaUaa.......................................................................   2        1    seq
....................uuacccuguuacauugGagaa.......................................................................   1        1    seq
....................Nuacccuguuacauuguagaau......................................................................   37       1    seq
....................uuacccuAuuacauuguagaau......................................................................   19       1    seq
....................uuacccuguuacauuguagaau......................................................................   121552   0    seq
....................uNacccuguuacauuguagaau......................................................................   3        1    seq
....................uuacccuguuacaGuguagaau......................................................................   3        1    seq
....................uuacccuguuacauuguUgaau......................................................................   6        1    seq
....................uuUcccuguuacauuguagaau......................................................................   5        1    seq
....................uuacccuguuacGuuguagaau......................................................................   11       1    seq
....................uuaccNuguuacauuguagaau......................................................................   8        1    seq
....................uuaccGuguuacauuguagaau......................................................................   2        1    seq
....................uuacccugGuacauuguagaau......................................................................   6        1    seq
....................uuacccuguuaGauuguagaau......................................................................   6        1    seq
....................uuacccuguuacauuguGgaau......................................................................   28       1    seq
....................uuacccuguuacauGguagaau......................................................................   2        1    seq
....................uuacccuUuuacauuguagaau......................................................................   8        1    seq
....................uuacccuguuacauugCagaau......................................................................   6        1    seq
....................uuacAcuguuacauuguagaau......................................................................   102      1    seq
....................uAacccuguuacauuguagaau......................................................................   86       1    seq
....................uuacccuguuacUuuguagaau......................................................................   9        1    seq
....................uuaAccuguuacauuguagaau......................................................................   22       1    seq
....................uuacccuguuacauuUuagaau......................................................................   6        1    seq
....................uuacccuguuUcauuguagaau......................................................................   13       1    seq
....................uuacccuguCacauuguagaau......................................................................   18       1    seq
....................uuacccuguuacauuguagaaG......................................................................   98       1    seq
....................uuacccuguAacauuguagaau......................................................................   43       1    seq
....................uuacccuguuacaCuguagaau......................................................................   14       1    seq
....................uuacccuguuacauuguagGau......................................................................   10       1    seq
....................uuaccAuguuacauuguagaau......................................................................   86       1    seq
....................uuGcccuguuacauuguagaau......................................................................   15       1    seq
....................uuCcccuguuacauuguagaau......................................................................   1        1    seq
....................uuacccuguuacauuguagaaA......................................................................   1268     1    seq
....................uuacccuguGacauuguagaau......................................................................   3        1    seq
....................uuacccAguuacauuguagaau......................................................................   56       1    seq
....................uuacccuguuacauuCuagaau......................................................................   10       1    seq
....................uuaGccuguuacauuguagaau......................................................................   2        1    seq
....................uuacGcuguuacauuguagaau......................................................................   5        1    seq
....................uuacccuguuacauuguagaGu......................................................................   13       1    seq
....................uuacccuguuacauugAagaau......................................................................   53       1    seq
....................uuacccuguuacauuguaUaau......................................................................   12       1    seq
....................Cuacccuguuacauuguagaau......................................................................   15       1    seq
....................uCacccuguuacauuguagaau......................................................................   15       1    seq
....................uuacccuguuaAauuguagaau......................................................................   47       1    seq
....................uuacccuguuacauuguagaUu......................................................................   10       1    seq
....................uuacccugCuacauuguagaau......................................................................   6        1    seq
....................uuacccuguuacauuAuagaau......................................................................   46       1    seq
....................uuacUcuguuacauuguagaau......................................................................   40       1    seq
....................Auacccuguuacauuguagaau......................................................................   467      1    seq
....................uuacccuguuacauugGagaau......................................................................   2        1    seq
....................uuacccCguuacauuguagaau......................................................................   12       1    seq
....................uuacccuguuCcauuguagaau......................................................................   1        1    seq
....................uuacccuguuacauuguagaaC......................................................................   13       1    seq
....................Guacccuguuacauuguagaau......................................................................   28       1    seq
....................uuaccUuguuacauuguagaau......................................................................   61       1    seq
....................uuacccuguuacauuguaAaau......................................................................   26       1    seq
```

Mature Star

caauccucgccaucaauucuuuacccuguuacauuguagaaugcuuguuauuccuuuguauucucaauguacaagguauuaagaauuggagguugaauuacuuccggaaguc

....................uuacccuguuacauuguagaCu..	1	1	seq
....................uuacccuCuuacauuguagaau..	7	1	seq
....................uuacccuguuaUauuguagaau..	9	1	seq
....................uuacccuguuGcauuguagaau..	11	1	seq
....................uuacccuguuacaAuguagaau..	28	1	seq
....................uGacccuguuacauuguagaau..	2	1	seq
....................uuacccuguuacauCguagaau..	17	1	seq
....................uuacccuguuacauuguagaaN..	2	1	seq
....................uuacccuguuacauuguaCaau..	6	1	seq
....................uuacccuguuacCuuguagaau..	1	1	seq
....................uuacccuguuacauuguagCau..	1	1	seq
....................uuacccuguuacauAguagaau..	36	1	seq
....................uuacccGguuacauuguagaau..	6	1	seq
....................uuacccuguuacauuNuagaau..	2	1	seq
....................uuacccuguNacauuguagaau..	1	1	seq
....................uuaUccuguuacauuguagaau..	7	1	seq
....................uuacccugNuacauuguagaau..	1	1	seq
....................uuacccuguuacauuguagUau..	6	1	seq
....................uuacccugAuacauuguagaau..	38	1	seq
....................uuacccuguuacauuguagaNug...	1	1	seq
....................uuaUccuguuacauuguagaaug...	4	1	seq
....................uuacccuguuacauugCagaaug...	5	1	seq
....................uuacccugCuacauuguagaaug...	6	1	seq
....................uuacccuguuacauCguagaaug...	49	1	seq
....................uuaGccuguuacauuguagaaug...	4	1	seq
....................uuacccuguCacauuguagaaug...	9	1	seq
....................Guacccuguuacauuguagaaug...	18	1	seq
....................Cuacccuguuacauuguagaaug...	5	1	seq
....................uuacccuguuacauGguagaaug...	1	1	seq
....................uuacccGguuacauuguagaaug...	3	1	seq
....................uuacccuguuacauAguagaaug...	22	1	seq
....................uuacccuguuacauuguaAaaug...	16	1	seq
....................uuacccuguuacauuguaUaaug...	1	1	seq
....................uuacccuguuacauuguGgaaug...	10	1	seq
....................uuacUcuguuacauuguagaaug...	19	1	seq
....................uuacccuguuacauuguagaauN...	1	1	seq
....................uuacccuguuacauuguagaaCg...	4	1	seq
....................uuacccuguuGcauuguagaaug...	11	1	seq
....................uuacccuguuacauuUuagaaug...	3	1	seq
....................uuacccAguuacauuguagaaug...	31	1	seq
....................uuacccuguuacauuguagaaGg...	5	1	seq
....................uuacccuUuuacauuguagaaug...	4	1	seq
....................uuacccuguuacauuguagaaAg...	11	1	seq
....................uCacccuguuacauuguagaaug...	10	1	seq
....................uuacccuCuuacauuguagaaug...	2	1	seq
....................uuacccuguuacauuguaCaaug...	2	1	seq
....................uuacccuguuacauuCuagaaug...	2	1	seq
....................uuacccuguuacaCuguagaaug...	4	1	seq
....................uuacccugAuacauuguagaaug...	14	1	seq
....................uuacccuguGacauuguagaaug...	1	1	seq
....................uuacccuguuacauuguagaauA...	317	1	seq
....................uuacccuguuacauuguagaGug...	5	1	seq
....................uGacccuguuacauuguagaaug...	3	1	seq
....................uuacccuguuUcauuguagaaug...	11	1	seq
....................uuacccugGuacauuguagaaug...	3	1	seq
....................uuaccGuguuacauuguagaaug...	1	1	seq
....................uuacccuAuuacauuguagaaug...	7	1	seq
....................uuacccuguAacauuguagaaug...	13	1	seq
....................uuacccuguuacauuguagUaug...	8	1	seq
....................uAacccuguuacauuguagaaug...	32	1	seq
....................uuacccuguuacaAuguagaaug...	6	1	seq
....................uuaAccuguuacauuguagaaug...	4	1	seq
....................uuUcccuguuacauuguagaaug...	3	1	seq
....................uuaccNuguuacauuguagaaug...	1	1	seq
....................uuacccuguuacauuAuagaaug...	10	1	seq
....................uuacccuguuaGauuguagaaug...	2	1	seq
....................uuacccuguuacauuguCgaaug...	1	1	seq
....................uuaccAuguuacauuguagaaug...	34	1	seq
....................Nuacccuguuacauuguagaaug...	11	1	seq
....................uuacccuguuacauuguagaUug...	3	1	seq

```
                    Mature                                  Star
caauccucgccaucaauucuuuacccuguuacauuguagaaugcuuguuauuccuuuguauucucaauguacaagguauuaagaauuggagguugaauuacuuccggaaguc
....................uuacccuguuaUauuguagaaug.....................................................................    4       1   seq
....................uuacAcuguuacauuguagaaug.....................................................................    40      1   seq
....................uuacccCguuacauuguagaaug.....................................................................    7       1   seq
....................uuacccuguuacauuguagaaug.....................................................................    56160   0   seq
....................Auacccuguuacauuguagaaug.....................................................................    205     1   seq
....................uuGcccuguuacauuguagaaug.....................................................................    12      1   seq
....................uuacccuguuacauuguagGaug.....................................................................    3       1   seq
....................uuacccuguuacauugAagaaug.....................................................................    17      1   seq
....................uuacccuguuacauuguagaauU.....................................................................    467     1   seq
....................uuacccuguuaAauuguagaaug.....................................................................    17      1   seq
....................uuacccuguuacauuguagCaug.....................................................................    2       1   seq
....................uuaccUuguuacauuguagaaug.....................................................................    31      1   seq
....................uuacccuguuacauuguagaauC.....................................................................    2       1   seq
....................uuacccuguuacUuuguagaaug.....................................................................    5       1   seq
....................uuacccuguuacauuguUgaaug.....................................................................    5       1   seq
....................uuacccuguuacGuuguagaaug.....................................................................    7       1   seq
....................uuacccuguuacauuguGgaaugc....................................................................    5       1   seq
....................uuacccuguuacauuguagUaugc....................................................................    7       1   seq
....................uuacccuguuacauuguagaGugc....................................................................    5       1   seq
....................uuacccuguuacauuguagaaGgc....................................................................    2       1   seq
....................uuacccuguuacauuguagaaugG....................................................................    18      1   seq
....................uuacccuguuacauuguagaaAgc....................................................................    11      1   seq
....................uuacccuguuaUauuguagaaugc....................................................................    15      1   seq
....................uuacccuguuacauAguagaaugc....................................................................    5       1   seq
....................uGacccuguuacauuguagaaugc....................................................................    1       1   seq
....................uuGcccuguuacauuguagaaugc....................................................................    1       1   seq
....................uuacccuguCacauuguagaaugc....................................................................    8       1   seq
....................uuacccuguuacauugGagaaugc....................................................................    1       1   seq
....................uuacccugCuacauuguagaaugc....................................................................    4       1   seq
....................uuacccuguuacaGuguagaaugc....................................................................    1       1   seq
....................uuacccuguuacauugAagaaugc....................................................................    8       1   seq
....................uuacccuguuacauuAuagaaugc....................................................................    7       1   seq
....................uuacccuguuacauuguagaaugN....................................................................    1       1   seq
....................uuacccuguuacauuguaAaaugc....................................................................    15      1   seq
....................Auacccuguuacauuguagaaugc....................................................................    135     1   seq
....................uuaGccuguuacauuguagaaugc....................................................................    2       1   seq
....................uuacccGguuacauuguagaaugc....................................................................    2       1   seq
....................uuacccuguuacaAuguagaaugc....................................................................    1       1   seq
....................uuacGcuguuacauuguagaaugc....................................................................    5       1   seq
....................uuacccuguuacauuguagaauUc....................................................................    2       1   seq
....................uuacccuguuacauuguagaaugU....................................................................    2583    1   seq
....................uuacccuguNacauuguagaaugc....................................................................    1       1   seq
....................uuacccuguuacauuguagaaugc....................................................................    34982   0   seq
....................uuNcccuguuacauuguagaaugc....................................................................    1       1   seq
....................uuacccuguuacauuguaCaaugc....................................................................    1       1   seq
....................uuacccuguuacauuguaUaaugc....................................................................    3       1   seq
....................uuacccuCuuacauuguagaaugc....................................................................    7       1   seq
....................uuacccuguuacauuguagGaugc....................................................................    8       1   seq
....................Nuacccuguuacauuguagaaugc....................................................................    10      1   seq
....................uuacccuguuacauuguagaaugA....................................................................    3679    1   seq
....................uuaAccuguuacauuguagaaugc....................................................................    9       1   seq
....................uuacccuguuacauGguagaaugc....................................................................    1       1   seq
....................uuacccAguuacauuguagaaugc....................................................................    18      1   seq
....................uuaccGuguuacauuguagaaugc....................................................................    1       1   seq
....................uNacccuguuacauuguagaaugc....................................................................    1       1   seq
....................uuacccuUuuacauuguagaaugc....................................................................    2       1   seq
....................uuaccAuguuacauuguagaaugc....................................................................    25      1   seq
....................uuaUccuguuacauuguagaaugc....................................................................    4       1   seq
....................uuaccUuguuacauuguagaaugc....................................................................    15      1   seq
....................uuacccuguuacauuguagaaCgc....................................................................    5       1   seq
....................uuacccuguuaGauuguagaaugc....................................................................    3       1   seq
....................uuacUcuguuacauuguagaaugc....................................................................    9       1   seq
....................uuacccuguuGcauuguagaaugc....................................................................    4       1   seq
....................uuUcccuguuacauuguagaaugc....................................................................    4       1   seq
....................uuacccuguuacGuuguagaaugc....................................................................    3       1   seq
....................uuacccuguuUcauuguagaaugc....................................................................    2       1   seq
....................uAacccuguuacauuguagaaugc....................................................................    20      1   seq
....................uuacccuguuaAauuguagaaugc....................................................................    12      1   seq
....................uuacccuguuacauuguUgaaugc....................................................................    2       1   seq
....................uuacccuguuacaCuguagaaugc....................................................................    5       1   seq
```

Mature **Star**

```
caauccucgccaucaauucuuuacccuguuacauuguagaaugcuuguuauuccuuuguauucucaauguacaagguauuaagaauuggagguugaauuacuuccggaaguc
....................uuacccuguuacauCguagaaugc....................................................................  3     1  seq
....................uuacccuguuacauuUuagaaugc....................................................................  2     1  seq
....................uuacccuguuacauugCagaaugc....................................................................  1     1  seq
....................uuacccuguuacauuguagaauAc....................................................................  2     1  seq
....................uuacccugAuacauuguagaaugc....................................................................  11    1  seq
....................uuacAcuguuacauuguagaaugc....................................................................  17    1  seq
....................uuacccuguAacauuguagaaugc....................................................................  14    1  seq
....................uCacccuguuacauuguagaaugc....................................................................  8     1  seq
....................uuacccuAuuacauuguagaaugc....................................................................  5     1  seq
....................uuacccCguuacauuguagaaugc....................................................................  1     1  seq
....................uuaccNuguuacauuguagaaugc....................................................................  2     1  seq
....................Cuacccuguuacauuguagaaugc....................................................................  2     1  seq
....................uuacccuguuacauuguagaUugc....................................................................  3     1  seq
....................Guacccuguuacauuguagaaugc....................................................................  18    1  seq
....................uuacccugGuacauuguagaaugc....................................................................  3     1  seq
....................uuacccuguuacUuuguagaaugc....................................................................  2     1  seq
....................uuaccUuguuacauuguagaaugcu...................................................................  4     1  seq
....................uuacccuguuacaAuguagaaugcu...................................................................  1     1  seq
....................uuacccuguuacauuguUgaaugcu...................................................................  2     1  seq
....................uuacccuguuacauuguagaaugcA...................................................................  3312  1  seq
....................uuacccuguuacauuguagaaugAu...................................................................  1     1  seq
....................Auacccuguuacauuguagaaugcu...................................................................  16    1  seq
....................uuacccuguuacauuguagaaugcG...................................................................  86    1  seq
....................uuacccugAuacauuguagaaugcu...................................................................  1     1  seq
....................uuacccuguuacauuguagaUugcu...................................................................  2     1  seq
....................uuGcccuguuacauuguagaaugcu...................................................................  1     1  seq
....................Nuacccuguuacauuguagaaugcu...................................................................  2     1  seq
....................uuacccuguuacaCuguagaaugcu...................................................................  1     1  seq
....................uCacccuguuacauuguagaaugcu...................................................................  1     1  seq
....................uuacUcuguuacauuguagaaugcu...................................................................  2     1  seq
....................uuacccAguuacauuguagaaugcu...................................................................  2     1  seq
....................uuacccuguuacauuguagaaugcu...................................................................  4488  0  seq
....................uuacccuguuacauugAagaaugcu...................................................................  2     1  seq
....................uuacccuguuacauuguagaaugUu...................................................................  1     1  seq
....................uuacccuguuacauAguagaaugcu...................................................................  3     1  seq
....................uuacccuguuacauuguagaGugcu...................................................................  1     1  seq
....................uuacccuUuuacauuguagaaugcu...................................................................  2     1  seq
....................uAacccuguuacauuguagaaugcu...................................................................  5     1  seq
....................uuacAcuguuacauuguagaaugcu...................................................................  1     1  seq
....................uuUcccuguuacauuguagaaugcu...................................................................  1     1  seq
....................uuacccuguuacauuguagaaNgcu...................................................................  1     1  seq
....................uuacccuguAacauuguagaaugcu...................................................................  1     1  seq
....................uuacccuguuacauuguaAaaugcu...................................................................  1     1  seq
....................uuacccuguCacauuguagaaugcu...................................................................  2     1  seq
....................uuacccuguuacauuguGgaaugcu...................................................................  2     1  seq
....................uuacccuguuacauuguagaauCcu...................................................................  1     1  seq
....................uuacccuguuacauuguagaaCgcu...................................................................  1     1  seq
....................uuaccAuguuacauuguagaaugcu...................................................................  2     1  seq
....................uuacccuguuacauuguagaaugcC...................................................................  108   1  seq
....................uuacccuguuaAauuguagaaugcu...................................................................  3     1  seq
....................uuacccuguuacauuguagaaugcuu..................................................................  489   0  seq
....................uuacccuguuacauuguagaaugcAu..................................................................  162   1  seq
....................uuacUcuguuacauuguagaaugcuu..................................................................  1     1  seq
....................uuacccugAuacauuguagaaugcuu..................................................................  1     1  seq
....................uuacccuguuacauuguagaaugcCu..................................................................  397   1  seq
....................uuacccuguuacauCguagaaugcuu..................................................................  1     1  seq
....................uuacccuguuacauuguagaaugcuA..................................................................  15    1  seq
....................uuacccuguuacauAguagaaugcuu..................................................................  1     1  seq
....................Auacccuguuacauuguagaaugcuu..................................................................  4     1  seq
....................uuacccAguuacauuguagaaugcuu..................................................................  1     1  seq
....................uuacccuguuacauuguagaaugUuu..................................................................  2     1  seq
....................uuacccuguuacauuguagaaugcuuU.................................................................  2     1  seq
.....................Gacccuguuacauuguaga........................................................................  1     1  seq
.....................uacccuguuacauuguaga........................................................................  38    0  seq
.....................uacccuguuacauuguagaa.......................................................................  5     0  seq
.....................uacccuguuacauuguagaau......................................................................  77    0  seq
.....................uacccuguuacauuguagUau......................................................................  1     1  seq
.....................Aacccuguuacauuguagaau......................................................................  2     1  seq
.....................uUcccuguuacauuguagaau......................................................................  1     1  seq
.....................uUcccuguuacauuguagaaug.....................................................................  2     1  seq
```

Mature Star

```
caauccucgccaucaauucuuuacccuguuacauuguagaaugcuuguuauuccuuuguauucucaauguacaagguauuaagaauuggagguugaauuacuuccggaaguc
.....................uacccugAuacauuguagaaug.....................................................................  1   1   seq
.....................uacccuguuacauuguagaaug.....................................................................  58  0   seq
.....................uacccuguuacauuguagaauU.....................................................................  11  1   seq
.....................uacccuguuacauuguagaaugA....................................................................  1   1   seq
.....................uacccuguuacauuguagaaugc....................................................................  6   0   seq
......................acccAguuacauuguagaa.......................................................................  1   1   seq
......................acccuguuacauuguagaau......................................................................  11  0   seq
......................acccuguuaAauuguagaau......................................................................  1   1   seq
......................acccAguuacauuguagaau......................................................................  1   1   seq
......................Gcccuguuacauuguagaaugc....................................................................  1   1   seq
......................acccuguuacauuguagaaugc....................................................................  64  0   seq
.......................cccuguuacauuguagaa.......................................................................  35  0   seq
........................ccuguuacauuguagaau......................................................................  18  0   seq
.........................cuguuacauuguagaaug.....................................................................  1   0   seq
.........................cuguuacauuguagaaugc....................................................................  1   0   seq
.............................uacaCuguagaaugcuugu................................................................  2   1   seq
...........................................................auucucaauguacaagguauu................................  13  0   seq
...........................................................auucucaauguacaagguauuaag.............................  4   0   seq
...........................................................auucucaauguacaagguauuaagU............................  1   1   seq
```
